# Circadian Rhythms Fluctuate the Treatment Effects of Intravesical Treatments on Rat Urinary Frequency Models

**DOI:** 10.1155/2024/6505595

**Published:** 2024-05-28

**Authors:** Tomofumi Watanabe, Takuya Sadahira, Yusuke Tominaga, Yuki Maruyama, Naoya Nagasaki, Takanori Sekito, Kohei Edamura, Toyohiko Watanabe, Motoo Araki, Masami Watanabe

**Affiliations:** Department of Urology, Okayama University Graduate School of Medicine, Dentistry and Pharmaceutical Sciences, Shikata-cho 2-5-1, Kita-ku, Okayama 700-8558, Japan

## Abstract

**Objectives:**

It is still not clear how the intravesical instillation of drugs affects rat urinary frequency. This study aimed to examine the dynamics of intravesical treatments' treatment effect on rat urinary frequency models by real-time and extended monitoring using a novel continuous urination monitoring system.

**Methods:**

Nine eleven-week-old female Wistar rats were divided into three groups to receive intravesical instillation of 0.1% acetic acid (AA), 1.0% AA, or phosphate-buffered saline (PBS). Thirty minutes later, these drugs were voided, and rats were moved to a continuous urination monitoring system, UM-100. UM-100 monitored rat urination quantitatively and continuously for 24 hours. Rats were then euthanized, and histopathologic examinations using a damage score validated the severity of bladder inflammation. We used nine additional rats to determine the treatment effect of various drugs against the urinary frequency. These rats were also treated with 1.0% AA in the same way and divided into three groups (*n* = 3 each) to receive intravesical instillation of lidocaine, silver nitrate (AgNO_3_), or dimethyl sulfoxide (DMSO), respectively. Thirty minutes later, rats were catheterized again and moved to the UM-100, and their voiding was monitored for 24 hours.

**Results:**

Intravesical instillation of AA increased the urinary frequency and decreased the mean voided volume (VV) in a concentration-dependent manner, with statistical significance at a concentration of 1.0% (urinary frequency; *p*=0.0007, mean VV; *p*=0.0032, respectively) compared with PBS. Histopathological analysis of these models demonstrated a significantly higher damage score of bladder mucosa in both 0.1% AA and 1.0% AA compared with PBS, with the severity in concordance with the clinical severity of urinary frequency (0.1% AA: *p* < 0.0001, 1.0% AA: *p* < 0.0001). Moreover, intravesical instillation of lidocaine, AgNO_3_, and DMSO decreased the urinary frequency. Continuous monitoring with UM-100 also demonstrated that the treatment effect of these intravesically instilled drugs occurred only at night.

**Conclusions:**

The extended monitoring of rat urination by UM-100 revealed a significant fluctuation in the treatment effect of intravesically instilled drugs between day and night. These findings may help establish novel therapies for urinary frequency.

## 1. Introduction

Urinary frequency and lower urinary tract symptoms (LUTSs) are common and negatively impact patient quality of life, with more than 60% prevalence in adult women [1–3]. Various treatments, including behavioral and drug therapy, have shown efficacy in treating LUTS [2, 4]. However, not all patients respond to these therapies, and there is an urgent need to develop more effective treatments for urinary frequency.

One promising approach for treating urinary frequency and LUTS is the intravesical administration of drugs. Intravesical instillation can directly deliver a higher concentration of drugs with minimal adverse events compared with whole-body administration. The intravesical instillation of several drugs/chemicals such as lidocaine, capsaicin, resiniferatoxin, silver nitrate (AgNO_3_), and dimethyl sulfoxide (DMSO) has already demonstrated some efficacy in treating overactive bladder, interstitial cystitis, bladder pain syndrome, and hemorrhagic cystitis [5–9]. However, the reported treatment effect of these drugs to date is less remarkable than expected, and a more effective technique for intravesical instillation of drugs, such as bladder indwelling devices, is needed [10].

Several methods have been tested in animal models, such as dogs and rodents, to examine the mechanisms of LUTS in humans and to establish novel treatments for LUTS. The voided stain on paper (VSOP) is one of the most common tests to evaluate mouse/rat LUTS noninvasively [11]. While this method provides many advantages, some have pointed out its limitations. For example, VSOP is a semiquantitative assay, and the recommended assay duration of the VSOP is limited to 2–4 hours [11]. Thus, the VSOP seems unfit for evaluating the effect of intravesical treatment, which requires accurate extended monitoring. Moreover, many variables influence VSOP assay outcomes (e.g., the filter paper used and its odor [11]). Thus, an alternative noninvasive method is required to overcome these limitations.

UM-100 (Melquest, Toyama, Japan) is a novel continuous urination monitoring tool for rodents that can record rat urination quantitatively and automatically every second for over 24 hours, similar to a previously reported human urinary monitoring system [12]. Due to its weight-based, real-time, and quantitative measurement method, UM-100 can potentially replace the VSOP and improve the accuracy of the assessment of rat lower urinary function in combination with the VSOP. In this study, we established rat LUTS models by intravesical instillation of acetic acid (AA) and examined the dynamics in the treatment effect of intravesical treatments on rat urinary frequency models by real-time and extended monitoring using UM-100 for the first time in the world.

## 2. Materials and Methods

### 2.1. Ethical Consideration

All animal experiments were conducted following the national guidelines and the relevant national laws on the protection of animals. Experimental protocols required for the animal studies were approved by the Animal Experiment Committee at Okayama University. The approval number is OKU-2021235.

### 2.2. Void Monitoring Using UM-100

**Figure 1:**
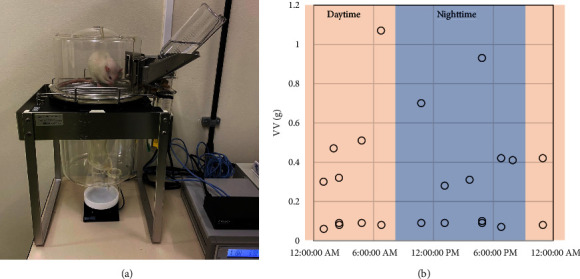
Experimental models with the UM-100. (a) Overview of the UM-100. (b) A representative analysis of data from the UM-100. UM-100 monitored the urination of an 11-week-old female Wistar rat for 24 hours (day and night were 12 hours each). The weight sensor under the metabolic cage detects urine drops, and UM-100 records each weight variation every second. UM-100 defines the beginning of a voiding event as a series of the weight changes, and the termination of the voiding event as no additional weight change in five seconds after that.

UM-100 (Melquest, Toyama, Japan) is a continuous monitoring system for rodent voiding behavior [13]. It comprises a metabolic cage manufactured by Techniplast (Buguggiate, Italy), a urine bag, a weight sensor that can measure weight variations in 10 mg increments, a central control unit, and a computer with software for data storage and analysis of the data. The weight sensor under the metabolic cage detects urine drops, and UM-100 records each weight variation every second. UM-100 defines the beginning of a voiding event as a series of weight changes and the termination of the voiding event as no additional weight change in five seconds after that (Figures 1(a) and 1(b)). The metabolic cage, urine collecting bags, and weight sensors can be expanded up to 4 units, and UM-100 can monitor up to 4 rodents simultaneously. After the examination, the mean voided volume (VV) and urinary frequency were calculated.

### 2.3. Rat Urinary Frequency Models

Eleven-week-old female Wistar rats were obtained from SLC, Inc. (Hamamatsu, Shizuoka, Japan).

**Figure 2:**
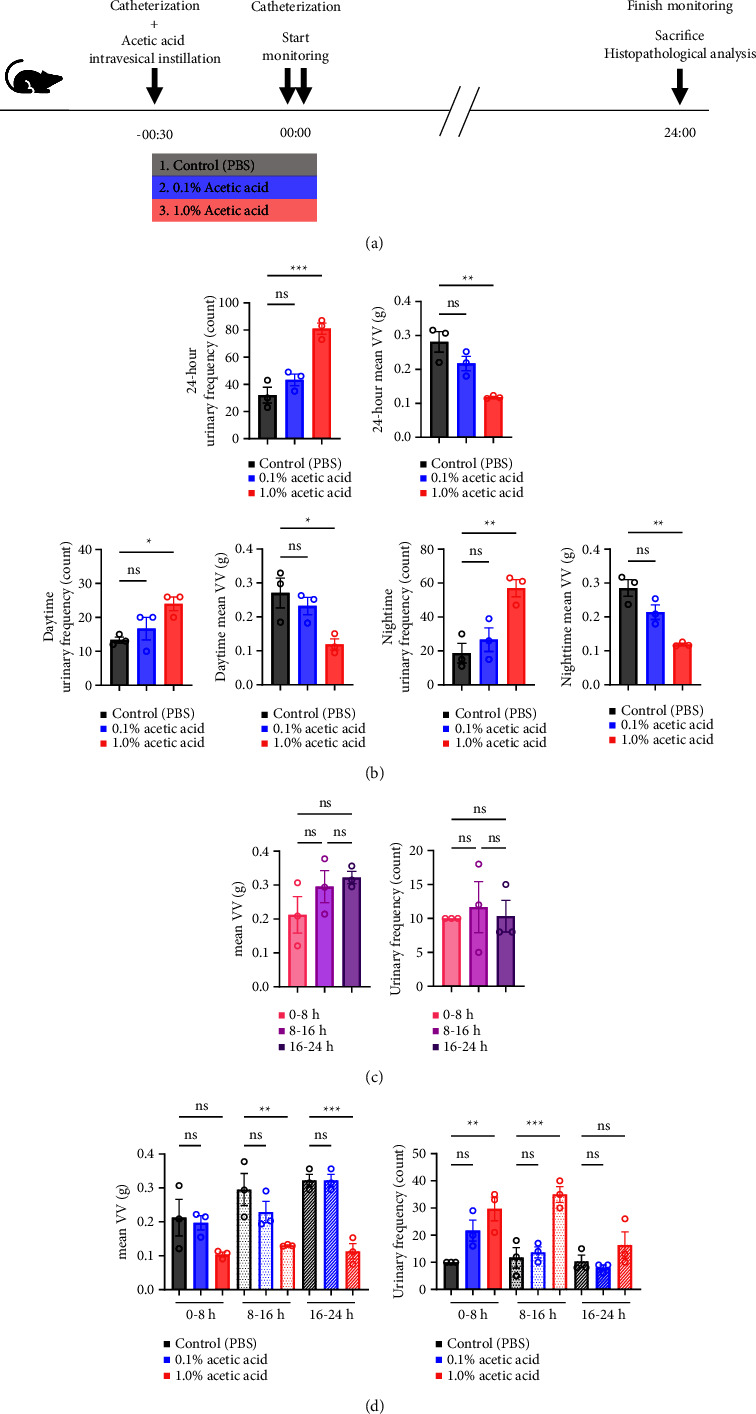
Acetic acid evokes bladder inflammation and urinary frequency. Nine rats were used to establish rat urinary frequency models. After the adaptation period for 24 hours in the cage, all rats were catheterized and divided into three groups (*n* = 3 each) to receive intravesical instillation of 300 *μ*L of 0.1% AA, 1.0% AA, or phosphate-buffered saline (PBS) as a negative control. Thirty minutes later, the rats were catheterized again and moved to the UM-100. Urination was monitored for 24 hours. Day and night were 12 hours each. (a) Experimental protocol. (b) Effect of AA on voiding behavior and its diurnal variation. Summary data of the mean voided volume (VV, left) and urinary frequency (right) over 24 hours (upper), day (lower left), and night (lower right) are shown. (c) Voiding behavior of rats in three periods during monitoring. Rats were catheterized, and 300 *μ*L of PBS was intravesically instilled. Thirty minutes later, the rats were catheterized again and moved to the UM-100. Urination was monitored for 24 hours. Summary data of the mean VV (left) and urinary frequency (right) in the indicated periods are shown. (d) Influence of time course during the experiment on the effect of AA. Summary data of the mean VV (left) and urinary frequency (right) in the indicated periods are shown. One-way ANOVA with the Bonferroni correction was used. Bars, mean; error bars, standard error of the mean; open circles, individual measurements; ns, not significant; ^*∗*^, *p* < 0.05; ^*∗∗*^, *p* < 0.01; ^*∗∗∗*^, *p* < 0.001.

**Figure 3:**
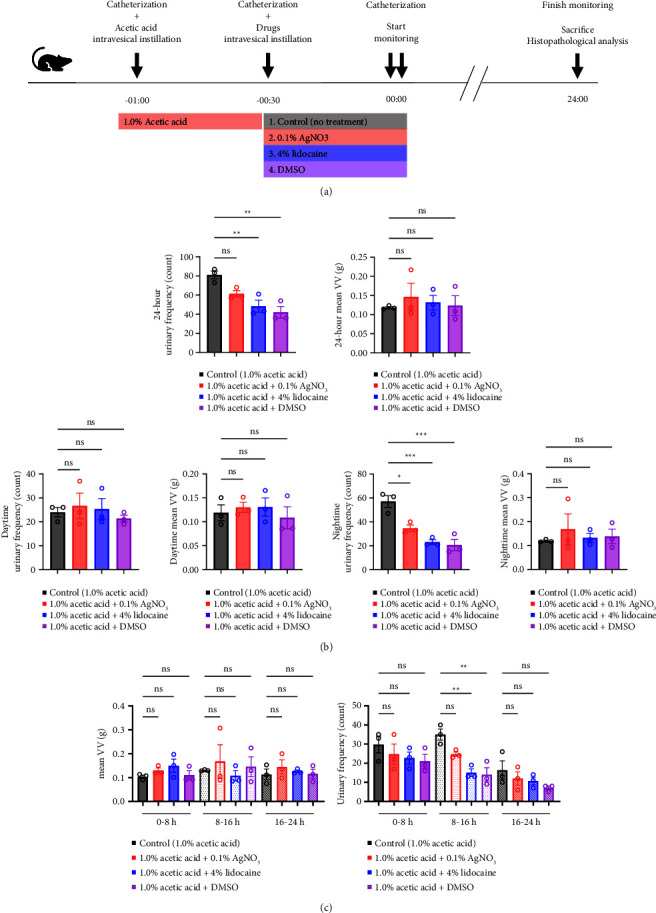
Intravesical instillation of drugs can relieve the urinary frequency of rat urinary frequency models at night. An additional nine rats were used to determine the treatment effect of various drugs against urinary frequency. These rats were treated with 1.0% AA in the same way as Figure 2 and subsequently divided into three groups (*n* = 3 each) to receive intravesical instillation of drugs (0.1% AgNO_3_, 4% lidocaine, or DMSO). Thirty minutes later, rats were catheterized again and moved to the UM-100, and their voiding was monitored for 24 hours. Day and night were 12 hours each. (a) Experimental protocol. (b) Effect of intravesical treatment on voiding behavior and its diurnal variation. Summary data of the mean voided volume (VV, left) and the urinary frequency (right) over 24 hours (upper), day (lower left), and night (lower right) are shown. (c) Influence of time course during the experiment on the effect of various drugs. Summary data of the mean VV (left) and urinary frequency (right) in the indicated periods are shown. One-way ANOVA with the Bonferroni correction was used. Bars, mean; error bars, standard error of the mean; open circles, individual measurements; ns, not significant; ^*∗*^, *p* < 0.05; ^*∗∗*^, *p* < 0.01; ^*∗∗∗*^, *p* < 0.001.

We used nine rats to establish rat urinary frequency models. After the adaptation period for 24 hours in the cage, all rats were catheterized and divided into three groups (*n* = 3 each) to receive intravesical instillation of 300 *μ*L of 0.1% AA, 1.0% AA, or phosphate-buffered saline (PBS) as a negative control, respectively [14, 15]. Thirty minutes later, the rats were catheterized again and moved to the UM-100. Urination was monitored for 24 hours. When we focused on the circadian rhythm, the data were analyzed with the monitoring period fractionated into two, i.e., daytime and nighttime, which were 12 hours each; daytime was from 8 AM to 8 PM, while nighttime was from 8 PM to 8 AM. When we focused on the elapsed time, we analyzed the data into three periods as follows: 0–8 hours, 8–16 hours, and 16–24 hours. Before and during the experiment, rats had access to food and drinking water *ad libitum*. After the experiment, rats were euthanized, and their bladders were subjected to histopathological analysis to validate the severity of inflammation (Figure 2(a)). We used an additional nine rats to determine the treatment effect of various drugs against urinary frequency. These rats were also treated with 1.0% AA in the same way and subsequently divided into three groups (*n* = 3 each) to receive intravesical instillation of drugs: 0.1% AgNO_3_, 4% lidocaine, or DMSO, respectively [16–18]. Thirty minutes later, rats were catheterized again and moved to the UM-100, and their voiding was monitored for 24 hours, as described above (Figure 3(a)).

### 2.4. Histopathological Analysis

Fixed tissues were processed routinely using standard techniques, subsequently embedded in paraffin, and sectioned into five *μ*m-thick sections stained with hematoxylin and eosin (HE). Histological changes in the bladder mucosa were assessed at 200x magnification under a BZ-X700 microscope (Keyence). In each specimen (*n* = 3 per treatment group), edema of the bladder mucosa in two randomly selected fields was evaluated using the following damage scoring system: 0 = normal (no edema), 1 = mild (no change in connective tissue thickness), 2 = moderate (connective tissue thickness increased by < 2fold), and 3 = severe (connective tissue thickness increased by >2 folds) as previously reported in rabbit cystitis models [19].

### 2.5. Statistical Analysis

All quantitative data were presented as bar graphs with the means ± standard error of the mean (SEM). The relationships between continuous variables among groups were compared using one-way ANOVA. For multiple tests, Bonferroni corrections were used. All tests were two tailed, and a *p* value <0.05 was considered to indicate statistical significance. All statistical analyses were performed using PRISM 9.3 software (GraphPad Software), Excel, and EZR version 1.61 (Saitama Medical University, Saitama, Japan). [20].

## 3. Results

### 3.1. Rat Urinary Frequency Models can be Established by Intravesical Instillation of Acetic Acid

**Figure 4:**
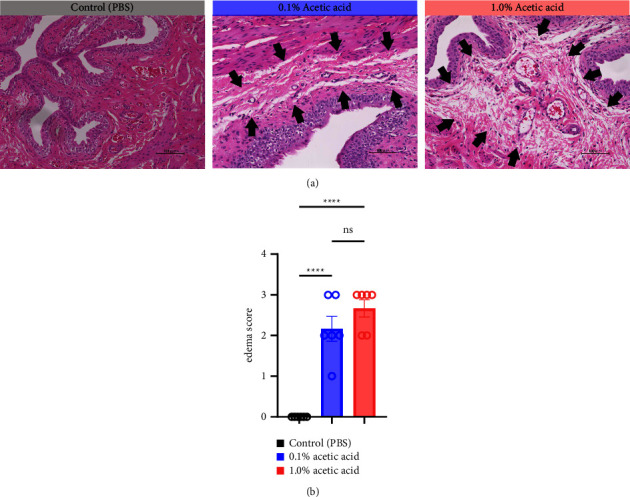
Histopathological analysis of bladder inflammation by AA. After monitoring, rats were euthanized, and their bladders were fixed and processed routinely and embedded in paraffin. Five *μ*m-thick sections were stained with hematoxylin-eosin (HE). (a) Representative histopathological images for rats treated with phosphate-buffered saline (PBS, left, no edema), 0.1% acetic acid (AA, middle, moderate connective tissue thickness increase), and 1.0% AA (right, heavy connective tissue thickness increase) are shown. Black arrows indicate stromal edema of the bladder mucosa. (b) The quantification of the bladder inflammation using the damage scoring system. Summary data of the damage score are shown. Bars, mean; error bars, standard error of the mean; open circles, individual measurements; ns, not significant; ^*∗∗∗∗*^, *p* < 0.0001.

Since intravesical instillation of AA facilitates micturition in rats, we used AA to establish rat urinary frequency models and monitored the voiding behavior of these rats. As a result, intravesical instillation of 1.0% AA significantly increased the urinary frequency and decreased the mean voided volume (VV) compared with PBS, both in daytime and nighttime (24-hour urinary frequency; *p*=0.0007, daytime urinary frequency; *p*=0.0297, nighttime urinary frequency; *p*=0.0079, 24-hour mean VV; *p*=0.0032, daytime mean VV; *p*=0.0262, nighttime mean VV; *p*=0.0017, respectively) (Figures 2(b) and 2(c)). Similar trends were observed with 0.1% AA although the differences were not statistically significant (24-hour urinary frequency; *p*=0.2953, daytime urinary frequency; *p*=0.5245, nighttime urinary frequency; *p*=0.7597, 24-hour mean VV; *p*=0.1583, daytime mean VV; *p*=0.8256, nighttime mean VV; *p*=0.0775, respectively) (Figure 2(b)). The elapsed time from the initiation of the experiment slightly, but not significantly, affected the voiding behavior in rat models (Figure 2(c)). The mean VV increased as time passed from the beginning of the experiment. Also, AA affected the voiding behavior of rats throughout the experiment, with a similar tendency in all three time periods mentioned above (Figure 2(d)). Moreover, histopathological analysis of these models demonstrated increased stromal edema (black arrows) in an AA concentration-dependent manner, with the severity in concordance with the clinical severity of urinary frequency (Figure 4(a)). When we quantified the bladder inflammation using the damage scoring system (e.g., PBS = 0, 0.1% AA = 2, and 1.0% AA = 3 in Figure 4(a)), the histopathological severity of the bladder mucosa was significantly higher in both 0.1% AA and 1.0% AA compared with PBS (PBS vs. 0.1% AA: *p* < 0.0001, PBS vs. 1.0% AA: *p* < 0.0001) (Figure 4(b)). Intravesical instillation of 1.0% AA induced severer bladder inflammation than 0.1% AA. However, the differences were not statistically significant (*p*=0.3634) (Figure 4(b)).

Intravesical instillation of drugs can relieve urinary frequency in rat urinary frequency models, especially at night.

Next, we examined the treatment effect of intravesical administration of various drugs on urinary frequency (Figure 4(a)). Intravesical instillation of 4% lidocaine and DMSO significantly decreased the urinary frequency but did not affect mean VV (4% lidocaine urinary frequency; *p*=0.0060, DMSO urinary frequency; *p*=0.0020, 4% lidocaine mean VV; *p* > 0.9999, DMSO mean VV; *p* > 0.9999, respectively) (Figure 3(b)). Intravesical administration of 0.1% AgNO_3_ showed a numerical, but not statistically significant, change in urinary frequency (*p*=0.0798) (Figure 3(b)). When we focused on the elapsed time from the initiation of the experiment, similar trends were observed in all three time periods during the experiment. Intravesical instillation of DMSO, 4% lidocaine, and 0.1% AgNO_3_ (sorted by the numerical change in descending order) decreased the urinary frequency but did not affect the mean VV (Figure 3(c)). However, when we focused on the circadian rhythm of the rats, the treatment effect of 0.1% AgNO_3_, as well as 4% lidocaine and DMSO, was emphasized at night (AgNO_3_ urinary frequency; *p*=0.0101, 4% lidocaine urinary frequency; *p*=0.0007, DMSO urinary frequency; *p*=0.0005) (Figure 3(b)). In contrast, all these drugs failed to show any treatment effect during the day (AgNO_3_ urinary frequency; *p* > 0.9999, 4% lidocaine urinary frequency; *p* > 0.9999, DMSO urinary frequency; *p* > 0.9999,) (Figure 3(b)). The circadian rhythm of the rats did not affect their mean VV (AgNO_3_ daytime mean VV; *p* > 0.9999, 4% lidocaine daytime mean VV; *p* > 0.9999, DMSO daytime mean VV; *p* > 0.9999, AgNO_3_ nighttime mean VV; *p* > 0.9999, 4% lidocaine nighttime mean VV; *p* > 0.9999, DMSO nighttime mean VV; *p* > 0.9999) (Figure 3(b)). Collectively, intravesical instillation of 0.1% AgNO_3_, 4% lidocaine, and DMSO relieved rat urinary frequency, especially at night.

## 4. Discussion

In this study, we performed quantitative and real-time analysis of rat voiding behavior for as long as 24 hours using UM-100. The extended monitoring of the voiding behavior of rat urinary frequency models demonstrated significant changes in the treatment effect of intravesically instilled drugs according to the circadian rhythm of the rats.

In our data, in accordance with previous reports, the intravesical instillation of AA evoked bladder inflammation that resulted in increased urinary frequency in rats, and intravesical instillation of lidocaine and DMSO relieved rat urinary frequency, while the treatment effect of silver nitrate was not as remarkable as other drugs [14, 21–23]. This result reflected the treatment effect of these drugs in patients with urinary frequency. Since intravesical instillation of silver nitrate itself often results in damage to the urothelium and scarring of the bladder, it has not been regarded as a promising intravesical instillation drug as lidocaine and DMSO [24]. Also, in this study, damage to the urothelium may have hindered the treatment effect of AgNO_3_ and failed to show statistical significance.

Moreover, our results support those of previous reports that noted a diurnal variation in the voiding behavior of rats and, more importantly, revealed that the treatment effect of intravesically instilled drugs is more significant at night [25, 26]. At night, rats are reportedly more active, have lower bladder capacity, and urinate more often [25]. Thus, our results indicate that intravesical instillation of drugs is more effective at night when bladders are more active and, therefore, more sensitive to external stimuli than during the day. This theory is consistent with another study, which found that intravesical treatments with b-FGF increased the urinary frequency only at night [26]. Since conventional experimental methods, such as VSOP and cystometry, are recommended to be performed for a relatively short duration, experiments conducted during the day may miss the treatment effect of drug candidates, which might show efficacy at night [11]. Moreover, in the VSOP, changing the filter paper or moving to the VSOP cage may wake the rats and affect the analysis during the day. In this study, we achieved extended monitoring of rat urinary behavior for a longer time with minimal external stimuli using UM-100 to reveal the dramatic fluctuation of the treatment effects of intravesical treatments on rat urinary frequency models.

The extended monitoring of the voiding behavior of rat urinary frequency models could be helpful, especially in evaluating the treatment effect of intravesical administration of drugs and intravesical indwelling devices. Since intravesical administration of some medicines demonstrated efficacy in treating urinary frequency or bladder pain, several intravesical indwelling devices have been developed to prolong their treatment effect and were examined in animal models. However, most of those experiments were insufficient to predict effectiveness in humans but remained preliminary, such as plasma concentrations of tested drugs and cystometry under general anesthesia [27, 28]. Our results indicate that durable monitoring of voiding behavior in conscious and nonrestrained rats has the potential to provide a new perspective on animal experiments to develop a more effective intravesical treatment for LUTS and urinary frequency.

Our study has several limitations. First, we could not directly compare the accuracy of the UM-100 and the VSOP. Second, we could not examine the validity and reproducibility of the UM-100 as reported for the VSOP [29]. Also, we could not evaluate the sexual difference in the effect of AA and drugs. In this study, we used female rats for the following two reasons: the prevalence of urinary incontinence or LUTS is higher in women than men and female rats are easier to catheterize than male [30, 31]. The results might be different if we examined male rats. Moreover, in this study, AA exposure was 30 minutes long in the same way as a previous report using bladder pain rat models [32]. A notable loss of urothelial integrity was not observed, and the treatment effect might have been different if these experiments had been performed under more severe bladder inflammation conditions. Finally, we could not examine the impact of multiple AA instillations to establish chronic cystitis models. Despite these limitations, our study is valuable in performing a quantitative and real-time analysis of rat voiding behavior for as long as 24 hours and demonstrating the significant changes in the treatment effect of intravesically instilled drugs according to the circadian rhythm of the rats.

## 5. Conclusion

In this study, we achieved a quantitative and real-time analysis of rat voiding behavior for as long as 24 hours using UM-100 and demonstrated the significant changes in the treatment effect of intravesically instilled drugs according to the circadian rhythm of the rats. These findings may help establish novel therapies for urinary frequency.

## Data Availability

The datasets generated and/or analyzed during the current study are available from the corresponding author on reasonable request.
